# Highly Sensitive Refractive Index Sensor Based on Adiabatically Tapered Microfiber Long Period Gratings

**DOI:** 10.3390/s131014055

**Published:** 2013-10-17

**Authors:** Wen Bin Ji, Swee Chuan Tjin, Bo Lin, Choong Leng Ng

**Affiliations:** 1 School of Electrical and Electronic Engineering, Optimus, Nanyang Technological University, Singapore 639798, Singapore; E-Mails: esctjin@ntu.edu.sg (S.C.T.); ngch0082@e.ntu.edu.sg (C.L.N.); 2 Institute for Infocomm Research, 1 Fusionopolis Way, #21-01 Connexis, Singapore 138632, Singapore; E-Mail: linb0007@e.ntu.edu.sg

**Keywords:** long period gratings, microfiber, refractive index sensor, adiabatic taper

## Abstract

We demonstrate a refractive index sensor based on a long period grating (LPG) inscribed in a special photosensitive microfiber with double-clad profile. The fiber is tapered gradually enough to ensure the adiabaticity of the fiber taper. In other words, the resulting insertion loss is sufficiently small. The boron and germanium co-doped inner cladding makes it suitable for inscribing gratings into its tapered form. The manner of wavelength shift for refractive indices (RIs) differs from conventional LPG, and the refractive index detection limit is 1.67 × 10^−5^.

## 1. Introduction

In the past a few decades, long-period fiber gratings (LPFG) have been broadly investigated for both communication [1] and sensing applications [2]. Traditional long period gratings (LPGs) are inscribed in a germanium (Ge)-doped fiber or a hydrogen-loaded single-mode fiber. Compared with fiber Bragg gratings (FBGs), LPGs are fabricated using an amplitude mask instead of an expensive phase mask, thus making the overall cost of LPGs sensing system advantageous to a certain extent. Apart from the amplitude mask method, there are also other techniques for fabricating LPGs in optical fibers such as lithography method which has been reported recently [3,4], or using a CO_2_ laser [5] to create periodic indentations on the fiber surface, and using a femtosecond laser to create refractive index variations [6]. These techniques will cause physical deformation in the fiber.

Recently, tapered fibers have attracted increasing attention since they have large evanescent fields and low-loss interconnection with single-mode fibers [7]. These fibers can be used for optical data storage [8] or filters [9], and they were also found to be popular in sensing applications including acceleration [10], temperature [11], and external refractive index (RI) [12–14] sensing. In particular for RI sensors, researchers have combined LPGs and tapered fibers together in order to increase the sensitivity [5,15].

In this work, we present long period gratings fabricated in a tapered photosensitive fiber using a KrF excimer laser. The fiber has double claddings and the inner cladding also has photosensitivity. The fiber was tapered adiabatically before writing the gratings and the resulting loss is within 1 dB. To the best of our knowledge, there has been no report on LPGs written in such a type of tapered fiber. We noted that for such type of microfiber long period grating (MLPG) sensor, the resonant wavelength of the LPG shifts to the longer wavelength when the external RI increases, in contrast to the normal LPG where the resonant wavelength shifts in the opposite direction to the shorter wavelength. We have successfully fabricated two such long period gratings with taper waist diameters of 60 μm and 55 μm. The sensitivity achieved in the RI region around 1.37 is around 600 nm/RIU which represents a significant improvement compared to normal LPG RI sensors.

**Figure 1.**
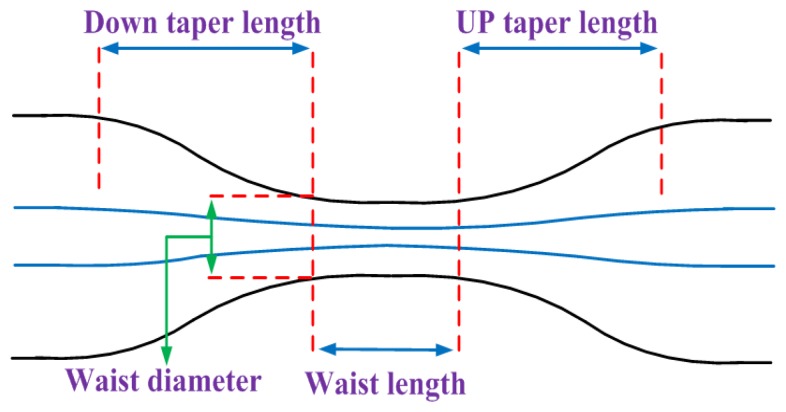
Schematic view of taper profile.

The criterion for adiabaticity of fiber taper is derived from the physical argument that taper length-scale must be much larger than the coupling length between the fundamental mode and the dominant coupling mode for power loss to be small [16]. As illustrated in Figure 1, the taper angle is defined as:

(1) $$\Omega (z)=\tan^{-1}\left(\frac{d\rho (z)}{dz}\right)$$

where ρ(z) is the radius along the z axis. In this criterion, the taper angle is assumed to be sufficient small so that:

(2) $${\text{z}}_{t}\approx \frac{\rho }{\Omega }$$

Assuming the taper is axisymmetric, as is usually the case with a single fiber taper, then the fundamental LP_01_ mode can couple only to the modes with the same azimuthal symmetry, *i.e.*, to the higher order LP_0_*_m_* cladding modes. If we are aiming to minimize loss from the fundamental mode, it is obvious that coupling will be mainly to the higher-order mode with propagation constant that is closest to the fundamental mode, *i.e.*, the LP_02_ mode. The coupling length between the two modes is taken to be the beat length between the fundamental mode and higher-order mode, and takes the form:

(3) $${\text{z}}_{b}(z)=\frac{2\pi }{\beta_{1}(z)-\beta_{2}(z)}$$

where *β*_1_(*z*). and *β*_2_(*z*) are the propagation constants of the two modes.

When *z_t_* ≫ *z_b_*(*z*) everywhere along the taper, the coupling loss will be negligible and the fundamental mode will propagate nearly adiabatically, whereas significant coupling will occur if *z_t_* ≪ *z_b_*(*z*) This indicates that *z_t_* = *z_b_*(*z*) is a condition just in between adiabatic and lossy tapers. Therefore, the taper angle can be derived as:

(4) $$\Omega (z)=\frac{\rho (z)(\beta_{1}(z)-\beta_{2}(z))}{2\pi }$$

This criterion indicates that when the core diameter is reduced to a small amount, in order to maintain low loss taper, the taper angle also needs to be sufficient small by means of making the fiber taper vary gradually.

For normal LPGs, the phase matching condition of coupling power from higher order mode to fundamental mode is shown to be [17]:

(5) $$|\beta_{1}-\beta_{2}|=\frac{2\pi }{\Lambda }$$

where *β*_1_ and *β*_2_ are the propagation constants of the fundamental mode and higher order mode, Λ is the period of the gratings. It can be easily derived from Equation (5) that the resonant wavelength under this phase matching condition takes the form:

(6) $$\lambda_{\text{res}}=(n_{co}^{\text{eff}}-n_{\text{clad},m}^{\text{eff}})\Lambda$$

Once the external refractive index increases, the effective refractive index of the mth cladding mode $n_{\text{clad},m}^{\text{eff}}$ will increase thus resulting a shift of resonant dip towards shorter wavelength.

## 2. Experimental Section

**Figure 2.**
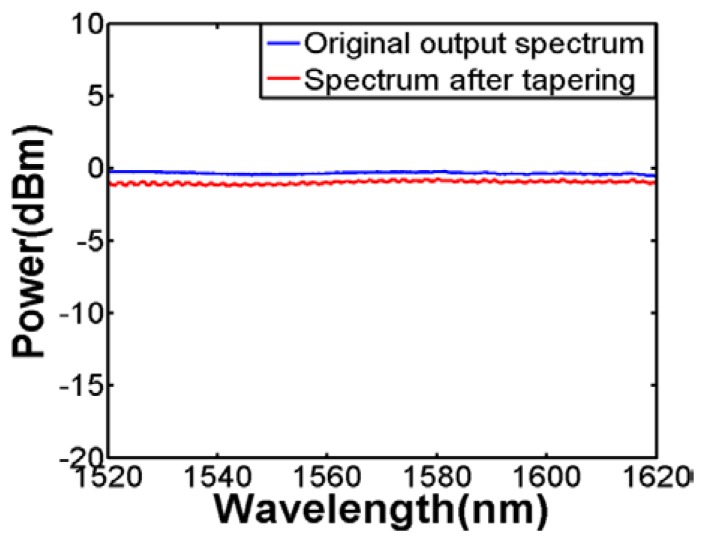
Transmission spectra of adiabatically tapered fiber (taper diameter: 60 μm).

There are several ways of obtaining fiber tapers. The conventional ways are by the flame brushing method whereby, using a fusion splicer or a CO_2_ laser to soften the fiber, coupled with a mechanical setup to stretch it. In our work, we have used a commercial fiber tapering machine (GPX-3000, Vytran, Morganville, NJ, USA) with motorized fiber holding stages. It is observed that the taper transition length, waist length, and waist diameter can be precisely controlled through this process so that the adiabaticity and uniformity can be ensured. An example of the transmission spectra before and after tapering is shown in Figure 2. The taper transition lengths are around 10 mm each and the taper waist length is around 20 mm. It can be seen that the loss is within 1 dB which is nearly negligible. More importantly, the flat response indicates that there is no higher-order excited mode. In this work, the fibers which are tapered to 60 μm and 55 μm were both adiabatically stretched in order to obtain a flat transmission spectrum. It is crucial to ensure the fiber is tapered gradual enough otherwise interference will occur as [18] demonstrates.

**Figure 3.**
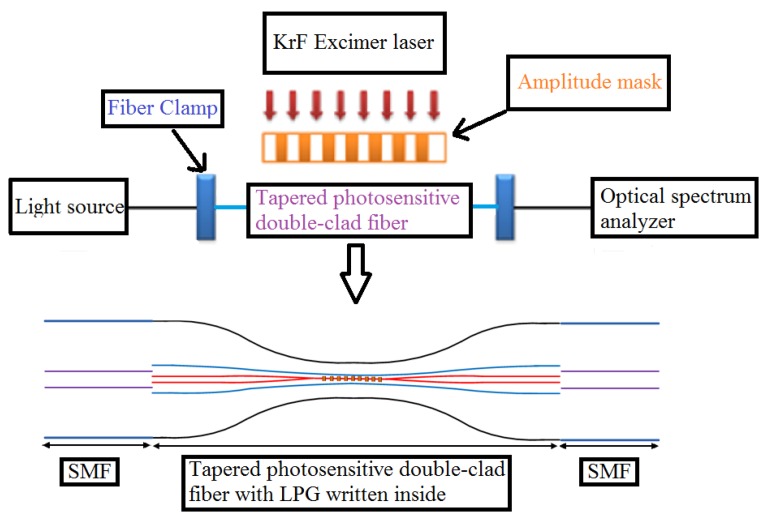
Schematic diagram of the experimental set-up.

After tapering, the fiber was exposed to a 248 nm KrF excimer laser with a pulse frequency of 10 Hz to fabricate the LPG. The periods of the amplitude mask for diameters of 60 μm and 55 μm are 320 μm and 450 μm, respectively. Figure 3 shows a schematic diagram of the LPG fabrication setup.

**Figure 4.**
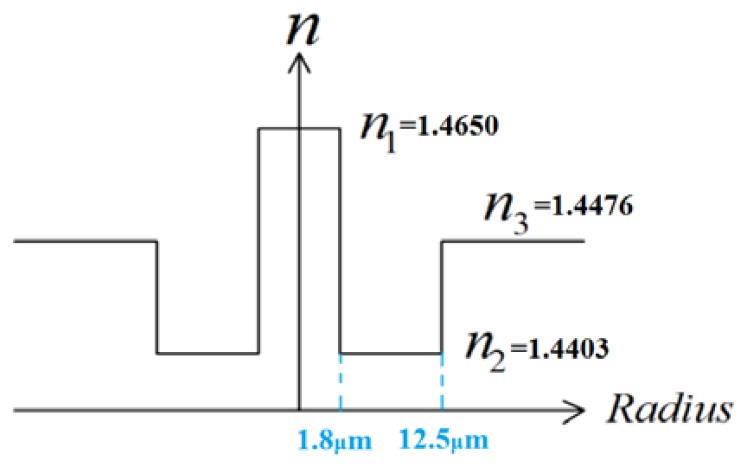
Refractive index profile of the W type double-clad fiber.

The single-mode fiber used in the experiment is the photosensitive double-clad fiber with High Ge-doped core (22 mol%) whose diameter is around 3.6 μm. The inner cladding has a diameter of 25 μm and is lightly doped with Ge and boron (B) (1 mol%). The co-doping of boron in germanosilicate fiber enhances the UV photosensitivity of the fiber [19,20]. The remaining area is the outer cladding with diameter of 125 μm. The refractive index distribution profile is W-shape as shown in Figure 4. The core has the highest RI while the inner cladding has the lowest. In the tapered fiber with waist diameter of 60 μm, the core diameter is reduced to 1.73 μm while the inner cladding diameter becomes 12 μm. This can be obtained by knowing that the core and inner cladding's sizes are changing proportionally with outer cladding [21], *i.e.*, n_core_ = 3.6 μm (original core diameter)× 60 μm (original inner cladding diameter)/125 μm (original outer cladding diameter) = 1.73 μm and n_ic_ = 25 μm × 60 μm/125 μm = 12 μm. To further enhance the photosensitivity, the tapered fibers were loaded with hydrogen at a temperature of 70 °C and a pressure of 2,000 psi for 192 h. The length of the gratings is 18 mm. The resonant dips of the two LPGs are observed at around irradiation of 2,000 pulses with fluence of 200 mJ/cm^2^. We stopped to adding more pulses until the resonant wavelength positions tended to stabilize. After the gratings were written, the fibers were annealed at 120 °C for 20 h to dispel the hydrogen that may still be present in the fiber.

## 3. Results and Discussion

**Figure 5.**
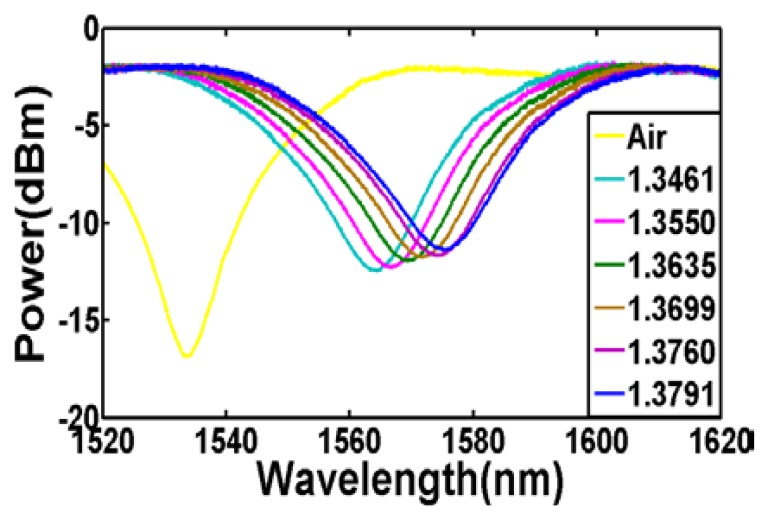
Wavelength response for different external refractive index (D = 60 μm).

To characterize the refractive index sensitivity of the sensor, the sensing region, which is the taper waist, was surrounded by a few droplets of salt solution. The salt solutions with different refractive index were prepared by diluting the saturated NaCl solution using distilled water. The refractive index of the solution was determined using a refractometer (KEM RA-130, Kyoto Electronics Manufacturing, Kyoto, Japan) with a resolution of 0.0001. The resulting wavelength responses are depicted in Figure 5. In contrast to the expected behavior of the transmission spectrum for conventional LPGs, we noted that the resonant dip of the fabricated LPG shifts towards longer wavelength when the external refractive index increases. The main reason for such phenomenon is due to the W-shaped refractive index profile of the fiber as discussed in the aforementioned section. After tapering, the original core diameter of 3.6 μm would have become smaller and does not contribute significantly to the core mode. In addition, it is believed that bulk of the Ge in the fiber core have dispersed into the inner cladding during the tapering process but hardly into the outer cladding [21]. In other words, for the tapered fiber, the effective refractive index of the core mode after tapering is lower than the refractive index of the outer cladding.

The phase matching condition for coupling between the higher-order modes to fundamental mode can still be derived from Equation (5), but the resonance wavelength is now given by:

(7) $$\lambda_{\text{res}}=(n_{\text{clad},m}^{\text{eff}}-n_{co}^{\text{eff}})\Lambda$$

According to the information provided by the manufacturer of the specialty fibre, the refractive index of the core is 1.4650, the inner cladding is 1.4403, and the outer cladding is 1.4476. The guiding mechanism after tapering was also simulated. It was found that such a tapered specialty fiber still supports single mode only. The mode field diameter of the new core mode is calculated to be 4.43 μm, which is much larger than the new core's diameter 1.73 μm, but much smaller than the new inner cladding's diameter 12 μm. Therefore, although the refractive index of the core is still much higher than the inner cladding and outer cladding, the effective index of the core mode will be greatly influenced by the inner cladding. As expected, the effective index of the core mode was calculated as 1.4443 which is smaller than refractive index of the cladding. This can justify the validity of Equation (7). For this simulation, we have no way to input the factors of how much Ge in the core is diffused to the inner cladding into consideration. Therefore, the value of the effective index 1.4443 actually will not be accurate enough. However, since after tapering the inner cladding size is much larger than the core size, the increase of the refractive index of the inner cladding caused by diffusion is nearly negligible. Now it can be seen from Equation (7) that the resonant wavelength will shift to a longer wavelength due to increase of external refractive index.

**Figure 6.**
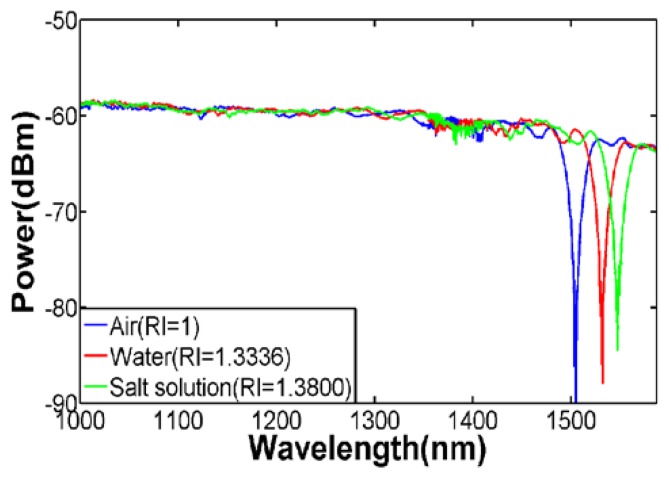
A broader spectrum for different external refractive index (D = 55 μm).

**Figure 7.**
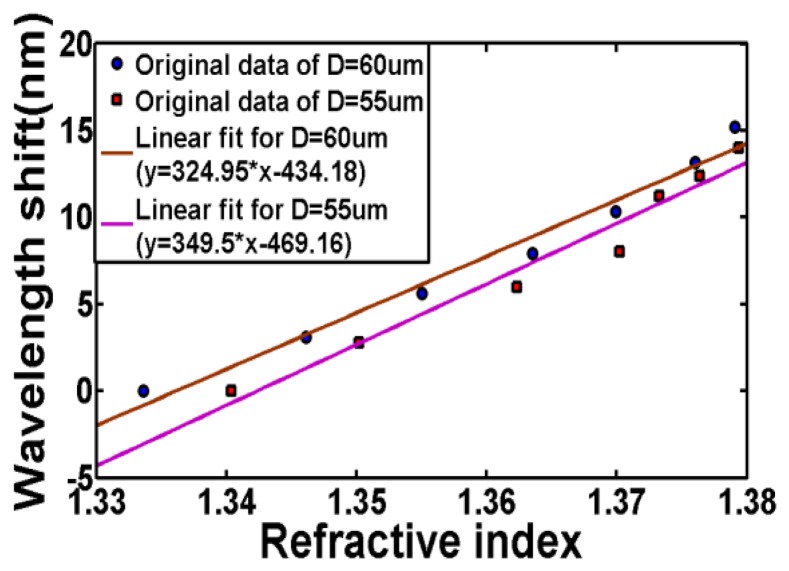
RI sensitivity characterization for both sensors (D = 60 μm and D = 55 μm).

A broader spectrum is shown in Figure 6, from which one can see that the couplings between the fundamental mode and the higher-order modes that have closer propagation constants with fundamental mode are very weak. This is because the power of the modes in the inner cladding is weak due to the higher refractive index of the outer cladding. The resonance dip become smaller as the index of liquid under test increases actually is not related to adiabaticity of the taper. For LPG, the transmission loss for the resonant wavelength will disappear when *n_ext_* = *n_cl_* since the core will see infinitely thick cladding region and without the cladding-surrounding interface, there is an absence of guided region and thus the cladding modes ceases to exist [22–24]. Therefore when the external refractive index gets closer to that of cladding, more power of cladding modes will be converted to power of radiation modes due to the lack of total internal reflection at the cladding-surrounding interface, resulting in a diminutive loss in the transmission spectrum. The maximum RI sensitivity achieved in our experiments is around 600 nm/RIU. With our OSA's resolution of 10 pm, the detection limit for refractive index is 1.67 × 10^−5^. Figure 7 illustrates the sensitivity characterization of two sensors with different taper diameter. The slope of the fiber with smaller outer diameter (55 μm), plotted using squares, and linearly fitted by purple line is slightly larger than the slope of the fiber with larger outer diameter, plotted using circles. As the diameter goes thinner, more evanescent field of the modes in outer cladding will penetrate into the surrounding thus the effective index of the outer cladding modes will be more easily impacted by the change of external refractive index. In another word, for same increment of external refractive index, the sensor with smaller diameter will have larger increment of effective index of the outer cladding modes. Therefore, the wavelength shift will be larger which can be obtained from Equation (7).

## 4. Conclusions

In summary, we have investigated long period gratings inscribed in an adiabatically tapered photosensitive specialty fiber with a W-shaped refractive index profile for refractive index sensing. The adiabaticity of the taper is achieved by making the fiber taper gradual enough. We have fabricated two MLPG sensors with different diameters and compared their sensitivity. The one with smaller diameter achieves a high RI resolution of 1.67 × 10^−5^ around the RI range of 1.38. The wavelength shift differs from conventional LPGs that the resonant wavelength shifts towards longer wavelength when external refractive index increases. Although the fiber is tapered into much a smaller diameter, the change in taper diameter is gradual enough to ensure the robustness of the fiber in terms of the mechanical strength. Therefore, such sensor has the potential for applications in environmental detection and biosensing areas.
